# Enhancement of pacing function by HCN4 overexpression in human pluripotent stem cell-derived cardiomyocytes

**DOI:** 10.1186/s13287-022-02818-y

**Published:** 2022-04-01

**Authors:** Yukihiro Saito, Kazufumi Nakamura, Masashi Yoshida, Hiroki Sugiyama, Satoshi Akagi, Toru Miyoshi, Hiroshi Morita, Hiroshi Ito

**Affiliations:** 1 Department of Cardiovascular Medicine, Okayama University Hospital, Okayama, Japan; 2 Department of Cardiovascular Medicine, Dentistry and Pharmaceutical Sciences, Okayama University Graduate School of Medicine, 2-5-1 Shikata-cho, 700-8558 Kita-ku, Okayama, Japan; 3 Department of Chronic Kidney Disease and Cardiovascular Disease, Dentistry, and Pharmaceutical Science, Okayama University Graduate School of Medicine, Okayama, Japan; 4 Department of Internal Medicine, Okayama Saiseikai General Hospital, Okayama, Japan; 5 Department of Cardiovascular Therapeutics, Dentistry and Pharmaceutical Sciences, Okayama University Graduate School of Medicine, Okayama, Japan

**Keywords:** Hyperpolarization-activated cyclic nucleotide-gated potassium channel 4, Human induced pluripotent stem cell-derived cardiomyocytes, Pacing

## Abstract

**Background:**

The number of patients with bradyarrhythmia and the number of patients with cardiac pacemakers are increasing with the aging population and the increase in the number of patients with heart diseases. Some patients in whom a cardiac pacemaker has been implanted experience problems such as pacemaker infection and inconvenience due to electromagnetic interference. We have reported that overexpression of HCN channels producing a pacemaker current in mouse embryonic stem cell-derived cardiomyocytes showed enhanced pacing function in vitro and in vivo. The aim of this study was to determine whether HCN4 overexpression in human induced pluripotent stem cell-derived cardiomyocytes (iPSC-CMs) can strengthen the pacing function of the cells.

**Methods:**

Human *HCN4* was transduced in the AAVS1 locus of human induced pluripotent stem cells by nucleofection and HCN4-overexpressing iPSC-CMs were generated. Gene expression profiles, frequencies of spontaneous contraction and pacing abilities of HCN4-overexpressing and non-overexpressing iPSC-CMs in vitro were compared.

**Results:**

HCN4-overexpressing iPSC-CMs showed higher spontaneous contraction rates than those of non-overexpressing iPSC-CMs. They responded to an HCN channel blocker and *β* adrenergic stimulation. The pacing rates against parent iPSC line-derived cardiomyocytes were also higher in HCN4-overexpressing iPSC-CMs than in non-overexpressing iPSC-CMs.

**Conclusions:**

Overexpression of HCN4 showed enhancement of *I*_f_ current, spontaneous firing and pacing function in iPSC-CMs. These data suggest this transgenic cell line may be useful as a cardiac pacemaker.

**Supplementary Information:**

The online version contains supplementary material available at 10.1186/s13287-022-02818-y.

## Introduction

The number of patients with bradyarrhythmia is increasing with the increase in the number of patients with heart diseases associated with aging. The treatment for bradyarrhythmia is mechanical pacemaker implantation, but there are remaining issues such as battery replacement due to battery drain, infection, electromagnetic interference and rate response. Biological pacemakers generated by gene transfer or cell transplantation are expected to be used as a new treatment for resolving these problems.

TBX18 gene transduction by an adenoviral vector has been shown to convert ventricular myocytes into sinoatrial node-like cells in vitro and in vivo. [1, 2] However, adenoviral vector-based gene therapy decays over time because adenoviral vectors are suitable for transient high expression and have immunogenicity that causes immune cells to eliminate reprogrammed cells. Therefore, this method is useful for temporary pacing but not for long-term use.

Several methods for the induction of sinoatrial node-like pacemaker cells derived from human pluripotent stem cells (PSCs) have been reported. Yechikov et al. [3] increased the induction efficiency of sinoatrial node-like cells from 10–15% to 20–30% by adding a TGFβ receptor inhibitor, SB431542, in addition to the Wnt inhibitor IWR1 after cardiac mesoderm induction during cardiac induction of human induced pluripotent stem cells (iPSCs). Protze et al. [4] found that NKX2-5-negative cells among cardiomyocytes induced by BMP4 and Activin A from human embryonic stem cells (ESCs) exhibited sinoatrial node-like gene expression patterns. Furthermore, they described a method in which 80% of the induced cardiomyocytes become sinoatrial node-like cells when BMP4, retinoic acid, and FGF receptor inhibitor (PD173074), and SB431542 were added in addition to a Wnt inhibitor during the cardiac mesoderm stage. Although this method is very useful, it is likely that the concentrations of various growth factors and inhibitors need to be adjusted for each cell line. Liu et al. [5] reported that the induction efficiency of sinoatrial node-like cells was 40–50% by adding BMP4, PD173074, and a retinoic acid receptor inhibitor, BMS189453, during the cardiac progenitor stage. Liang et al. [6] found that 70% of the induced cardiomyocytes were sinoatrial node-like cells by removing the Wnt inhibitor after mesoderm induction, but the cardiomyocyte yield was as low as 20%. Pezhouman et al. [7] reported that adjustments of the cell seeding density and the concentration of a GSK3 inhibitor, CHIR99021, during mesoderm induction resulted in the generation of NKX2-5-negative TBX5-positive cardiac progenitor cells that gave rise to podoplanin-positive sinoatrial node-like cells with high efficiency. However, no functional analysis has been performed. Zhao et al. [8] transduced *TBX3* with a lentiviral vector during the cardiac mesoderm stage, but the proportion of sinoatrial node-like cells was only 20%. For each method, there is room for improvement in induction efficiency, yield and simplicity.

Recently, designer cells with functions added by genetic modification, such as cells used in chimeric antigen receptor (CAR) T-cell therapy, are expected to become new tools for cell-based therapy [9, 10]. Previously, we have transduced *HCN4* into mouse embryonic stem cell-derived cardiomyocytes (ESC-CMs) to intensify their pacing function in vitro and in vivo [11, 12]. *HCN4* encodes a hyperpolarization-activated cyclic nucleotide-gated potassium channel (HCN channel) producing a pacemaker current. It has been reported that human PSC-derived cardiomyocytes (PSC-CMs) express HCN4 and have some pacing potency that would be lost along maturation [13–16]. HCN4 overexpression seems simpler than other methods to generate pacemaker cells from human PSCs, however, this strategy have not been tested with human PSCs. In addition, the electrophysiological properties are different between rodent cardiomyocytes and human cardiomyocytes [17]. In this study, we investigated whether HCN4 overexpression in human induced pluripotent stem cell-derived cardiomyocytes (iPSC-CMs) improves pacemaker function in vitro.

## Methods

### Method summary

**Fig. 1:**
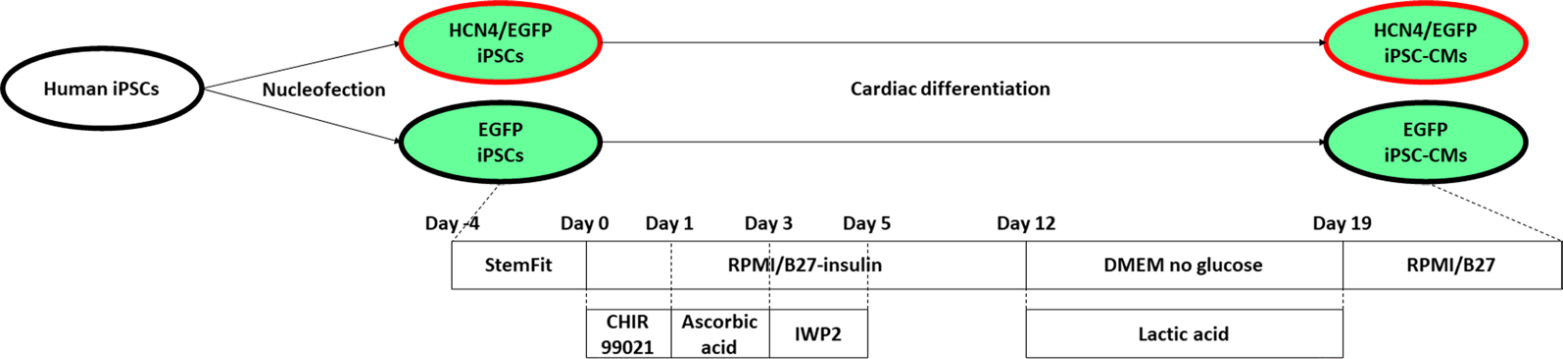
Method summary to generate HCN4-overexpressing human cardiomyocytes. iPSCs: induced pluripotent stem cells. iPSC-CMs: induced pluripotent stem cell-derived cardiomyocytes

The strategy to generate HCN4-overexpressing iPSC-CMs is summarized in Fig. 1.

### Donor plasmid vector construction

The sequences of “CAG promoter_IRES_*EGFP*_polyA” in pCAGIG (Cat#. 11159, Addgene, Watertown, MA, United states), “splice acceptor (SA)_loxP_PGK promoter_*neomycin resistance gene* (*Neo*)_loxP” in pRosa26-DEST (Cat#. 21189, Addgene) and “AAVS1 3' homology arm_*Ampicilline resistance gene* (*AmpR*)_AAVS1 5' homology arm” in AAV-CAGGS-EGFP (Cat#. 22212, Addgene) were amplified by the polymerase chain reaction (PCR) with PrimeSTAR GXL DNA polymerase (Cat#. R050A, Takara bio, Shiga, Japan), purified using a QIAquick PCR Purification Kit (Cat#. 28104, Qiagen, Hilden, Germany), and then ligated with an In-fusion HD enzyme (Cat#. 639648, Clontech, Mountain View, CA, United States) to get the control donor vector, “pAAVS1_SA_loxP_PGK_*Neo*_loxP/CAG_ IRES_*EGFP/AmpR*”. Human *HCN4* open reading frame (ORF) subcloned into pCMV6-XL5 vector (Cat#. SC303651) was purchased from Origene (Rockville, MD, United States). *HCN4* ORF was amplified by PCR, purified, and ligated with an Xho I-digested control donor vector to get “pAAVS1_SA_loxP_PGK_*Neo*_loxP/CAG_ HCN4_IRES_*EGFP/AmpR*”.

PCR primers are shown in Table 1.

**Table 1:** PCR primers

Name		Sequence	Product size (bp)	Annealing temperature (℃)
*Subcloning PCR*				
PGK-*Neo*	Forward	TGGGGCGGGATCTGTAGGGCGCAGTA	2886	64
	Reverse	AACTAGATAACTTCGTATAGCATACATTATACGAAGT		
AAVS1 homology arm/*Amp*^*R*^	Forward	ACCTGCAGACTAGGGACAGGATTGGT	5624	64
	Reverse	ACAGATCCCGCCCCACTGTGGGGTGGA		
CAG-IRES-*EGFP*	Forward	CGAAGTTATCTAGTTATTAATAGTAATCAATTACGGGG	3614	62
	Reverse	CCCTAGTCTGCAGGTCGAGGGATCT		
Human *HCN4*	Forward	CAAAGAATTCCTCGAGACCATGGACAAGCTGCCGCCGTC	3647	64
	Reverse	CCGCGATATCCTCGAGTCATAGATTGGATGGCAGTTTGGAGC		
*Flanking PCR*				
Primer 1		TGGGCTTTGCCACCCTATGCTGACA		64
Primer 2		ACAAGCAGAAGAACGGCATCAAGGTGAA		
Primer 3		ACCAACCATCCCTGTTTTCCTAGGACTGA		
*qPCR*				
*GAPDH*	Forward	CAACGACCACTTTGTCAAGCTC	144	60
	Reverse	TCTCTTCCTCTTGTGCTCTTGC		
*HCN4*	Forward	GGTGTCCATCAACAACATGG	66	60
	Reverse	GCCTTGAAGAGCGCGTAG		
*MYL2*	Forward	GTGCTGAAGGCTGATTACGTTC	121	60
	Reverse	TGTAGTCCAAGTTGCCAGTCAC		
*NKX2-5*	Forward	GTCCCCTGGATTTTGCATTCAC	100	60
	Reverse	ATAATCGCCGCCACAAACTCTC		
*NR2F2*	Forward	TCGCCTTTATGGACCACATACG	149	60
	Reverse	TTCCACATGGGCTACATCAGAG		
*SCN5A*	Forward	AGAAGATGGTCCCAGAGCAATG	131	60
	Reverse	AATCTGCTTCAGAACCCAGGTC		
*SHOX2*	Forward	ATCGCAAAGAGGATGCGAAAGGGA	86	60
	Reverse	TTCCAGGGTGAAATTGGTCCGACT		
*TBX3*	Forward	TTGAAGACCATGGAGCCCGAAGAA	91	60
	Reverse	CCCGCTTGTGAAACTGATCCCAAA		
*TBX18*	Forward	TTAACCTTGTCCGTCTGCCTGAGT	147	60
	Reverse	GTAATGGGCTTTGGCCTTTGCACT		
*TNNT2*	Forward	TTCACCAAAGATCTGCTCCTCGCT	166	60
	Reverse	TTATTACTGGTGTGGAGTGGGTGTGG		

### Cell culture

Human iPSCs generated from dermal fibroblasts obtained from a healthy subject with retroviral vectors were used in this study [11, 18]. The cells were dissociated with StemPro Accutase Cell Dissociation Reagent (Cat#. A1110501, Thermo Fisher Scientific, Waltham, MA, United States). Then they were resupended in StemFit AK02N (Cat#. AK02N, Ajinomoto Healthy Supply, Tokyo, Japan) with 10 µmol/L Y-27632 (Cat#. S1049, Selleck Chemicals, Houston, TX, United States) and seeded at 1.5 × 10^3^/cm^2^ on 0.25 µg/cm^2^ iMatrix-511 silk (Cat#. 892021, Matrixome, Osaka, Japan)-coated 6-well plates (Cat#. 3516, Corning, Corning, NY, United States) [19]. The medium was changed with StemFit without Y-27632 the next day and cells were passaged once a week.

### Nucleofection

Human iPSCs were harvested using StemPro Accutase Cell Dissociation Reagent. Ten µg of a non-linearized donor vector, 5 µg of AAVS1 1L TALEN (Cat#. 35431, Addgene) and 5 µg of AAVS1 1R TALEN (Cat#. 35432, Addgene) were transduced with a Nucleofector II/Program B-016 (Cat#. AAD-1001N, Amaxa Biosystems, Nordrhein-Westfalen, Germany) and an Amaxa Human Stem Cell Nucleofector Kit 1 (Cat#. VAPH-5012, Lonza, Basel, Switzerland). The cells were treated with 50 µg/ml G-418 (Cat#. 4727878001, Roche Applied Science, Penzberg. Germany) for 10 days. EGFP-positive clones were picked up and expanded on iMatrix-511-coated plates in StemFit AK02N. The established clones were genotyped by PCR. PCR primers are shown in Table 1.

### Cardiac differentiation of human iPSCs

Our cardiac differentiation protocol was based on that previously reported by Lian et al.[20]. Human iPSCs were dissociated into single cells with StemPro Accutase Cell Dissociation Reagent and seeded on 0.5 µg/cm^2^ iMatrix-511 silk-coated 12-well plates (Cat#. 3513, Corning) at 3 × 10^5^/cm^2^ in StemFit AK02N supplemented with 10 µmol/L Y-27632. The medium was changed with a medium without Y-27632 every day. Four days after plating, at day 0, cells were treated with 6 µmol/L CHIR99021 (Cat#. S2924, Selleck chemicals) in RPMI 1640 (Cat#. 11875093, Thermo Fisher Scientific) supplemented with B-27 supplement minus insulin (Cat#. A1895601, Thermo Fisher Scientific). After 24 h, at day 1, the medium was changed to RPMI supplemented with B-27 supplement minus insulin and 100 µmol/L ascorbic acid 2-glucoside (Cat#. AG121, Hayashibara, Okayama, Japan). On day 3, the medium was changed to RPMI supplemented with B-27 supplement minus insulin and 5 µmol/L IWP2 (Cat#. S7085, Selleck chemicals). On day 5, the medium was changed to RPMI supplemented with B-27 supplement minus insulin. From day 7, the medium was changed every 2 or 3 days. On day 12, the medium was changed to DMEM, no glucose (Cat#. 11966025, Thermo Fisher Scientific) supplemented with 16 mM L-( +)-Lactic acid (Cat#. L1750, Merck, Kenilworth, NJ, United States) to remove non-cardiomyocytes [21]. Until day 19, the medium was changed every 2 or 3 days. After cardiomyocyte purification, the cells were maintained in RPMI supplemented with B-27 supplement (Cat#. 17504044, Thermo Fisher Scientific).

### Quantitative PCR

Total RNA from iPSC-CMs was extracted using Trizol Reagent (Cat#. 15596026, Thermo Fisher Scientific) and PureLink RNA Mini Kit (Cat#. 12183018A, Thermo Fisher Scientific). Complementary DNA was synthesized from 500 ng of total RNA using SuperScript IV VILO Master Mix (Cat#. 11756050, Thermo Fisher Scientific) according to the manual and subjected to PCR amplification. PowerUp SYBR Green Master Mix (Cat#. A25742, Thermo Fisher Scientific) and Quantistudio 1 Real-Time PCR System (Cat#. QS1, Thermo Fisher Scientific) were used for quantitative PCR (qPCR). Quantitative PCR experiments were performed in duplicate. The qPCR data were processed by the ΔΔCT method. PCR primers are shown in Table 1.

### Transmission electron microscopy (TEM)

HCN4-overexpressing iPSC-CMs were seeded on a Matrigel-coated 35 mm dish. The cells were fixed by chemical fixation and TEM was performed by Tokai Electron Microscopy (Aichi, Japan).

### Immunofluorescence

The purified iPSC-CMs were plated on 0.5 µg/cm^2^ iMatrix-511 silk-coated plates, fixed in 4% paraformaldehyde (Cat #. 09154-85, Nakalai tesque, Kyoto, Japan), permeabilized with 0.1% Triton X-100/ phosphate-buffered saline (PBS), and blocked with 10% goat serum and 0.1% Triton X-100/ PBS (Cat#. G9023, Merck). The cells were stained overnight at 4℃ with primary antibodies againstα-actinin (1:1000 dilution, Cat#. A7811, Merck), cardiac Troponin T (1:16,000 dilution, Cat#. GTX28295, GeneTex, Irvine, CA, United States), HCN4 (1:250 dilution, Cat#. sc-58622, Santa Cruz Biotechnology, Dallas, TX, United States), NKX2-5 (1:2500 dilution, Cat#. 8792S, Cell Signaling Technology, Danvers, MA), and Connexin 43 (1:500 dilution, Cat#. 83649, Cell Signaling Technology). Secondary antibodies were iFluor™ 488 goat anti-mouse IgG (H + L), iFluor™ 555 goat anti-mouse IgG (H + L), iFluor™ 488 goat anti-rabbit IgG (H + L), iFluor™ 555 goat anti-rabbit IgG (H + L) (1:1000 dilution, Cat#. 16528, 16540, 16678, 16690, AAT Bioquest, Sunnyvale, CA, United States) and Alexa Fluor 555 goat anti-rat IgG (H + L) (1:1000 dilution, ab150158, Abcam, Cambridge, United Kingdom). Nucleus staining was performed with Hoechst 33,342 (1:10,000 dilution, Cat#. H3570, Thermo Fisher Scientific). Plasma membrane staining was performed with Wheat Germ Agglutinin, Alexa Fluor™ 350 Conjugate (Cat#. W11263, Thermo Fisher Scientific).

### Electrophysiology

The perforated patch‐clamp technique was performed using an Axopatch 200B amplifier (Molecular Devices, San Jose, CA, United States) and data were acquired with pClamp10.2/Clampfit (Molecular Devices). Cardiomyocytes were dissociated using TrypLE Select Enzyme (10X) (Cat#. A1217701) for 15 min, resuspended in RPMI 1640 supplemented with 20% fetal bovine serum and replated on Matrigel (Cat#. 354277, Corning)-coated cover glasses and incubated for 72 h. The cells were superfused with a bath solution containing (in mM): 132 NaCl, 4.8 KCl, 2.0 CaCl_2_, 1.2 MgCl_2_, 1.0 BaCl_2_, 2.0 MnCl_2_, 5.0 D-glucose, and 10 Hepes; pH 7.4. Pipettes (2–4 MΩ resistances) were filled with a pipette solution containing (in mM): 110 K-aspartate, 5.0 K_2_-ATP, 11 EGTA, 1.0 CaCl_2_, 1 MgCl_2_, and 5 Hepes; pH 7.2. Then 0.3 mg/mL Amphotericin B (Cat #. 02743–04, Nakalai tesque) was added to the pipette solution to achieve patch perforation (10–20 MΩ; series resistance). The *I*_f_ current was activated by a standard activation protocol. *I*_f_ currents through activated HCN channels were obtained during hyperpolarizing test pulses of 5 s between − 45 and − 125 mV in 20-mV increments from a holding potential of − 35 mV. Action potentials were measured using a modified Tyrode’s solution containing (in mM): 140 NaCl, 5.4 KCl, 1.8 CaCl_2_, 1.0 MgCl_2_, 5.5 glucose, and 5.0 HEPES; pH 7.4 (NaOH). The pipette solution contained (in mM) 110 DL-aspartic acid, 30 KCl, 1 CaCl_2_, 5 ATP-Mg, 5 Creatine P-Na, 5 HEPES, and 10 EGTA; pH 7.25 (KOH). To achieve patch perforation (series resistance: 10–20 MΩ), amphotericin B (0.3 mg/mL) was added to the pipette solution. The temperature was maintained at 35–36 °C by a TC-344B dual channel heating system (Warner Instruments, Holliston, MA, United States).

### Counting frequencies of spontaneous beating

The purified cardiomyocytes were plated on 0.25 µg/cm^2^ iMatrix-511 silk-coated plates at 1 × 10^5^/cm^2^ in RPMI 1640 with B-27 supplement and incubated for 4 days. We counted spontaneous eating frequencies for 15 s with an optical microscope and examined responses to 10 µmol/L ivabradine (Cat#. SML0281, Merck) and 1 µmol/L isoproterenol (Cat#. I5627, Merck).

### Counting contraction frequencies of EGFP-negative iPSC-CMs paced by EGFP-positive iPSC-CMs

The center of each well of 12-well plates was coated with 30 µL PBS containing 0.5 µg iMatrix-511 silk. Next day, 4 × 10^5^ EGFP-positive iPSC-CMs were resuspended in 30 µL RPMI 1640 with B-27 supplement, 10 µmol/L Y27632 and 5% fetal bovine serum (Cat#. F7524, Merck) and then seeded on the iMatrix-511-coated area (day 0). On day 1, 1 mL/well RPMI 1640 with B-27 supplement was added. On day 3, the medium was changed to RPMI 1640 with B-27 supplement and 1 µg/well iMatrix-511 silk. On day 4, 4 × 10^5^ EGFP-negative parent iPSC-derived cardiomyocytes were resuspended in 1 mL RPMI 1640 with B-27 supplement, 10 µmol/L Y27632 and 5% fetal bovine serum and seeded on 12-well plates containing EGFP-positive cardiomyocytes. On day 5, the medium was changed to RPMI 1640 with B-27 supplement. On day 7, synchronized beating rates for 15 s of EGFP-negative iPSC-CMs away from the EGFP-positive area were counted with an optical microscope.

### Statistical analysis

All data are expressed as means ± standard deviation. Statistical analysis was performed by Student’s t-test for unpaired data or one-way ANOVA with comparison of different groups by Dunnett’s post hoc test using SPSS ver. 24. Values of *P* < 0.05 were considered significant.

## Results

### Generation of HCN4-overexpressing human iPSCs

**Fig. 2:**
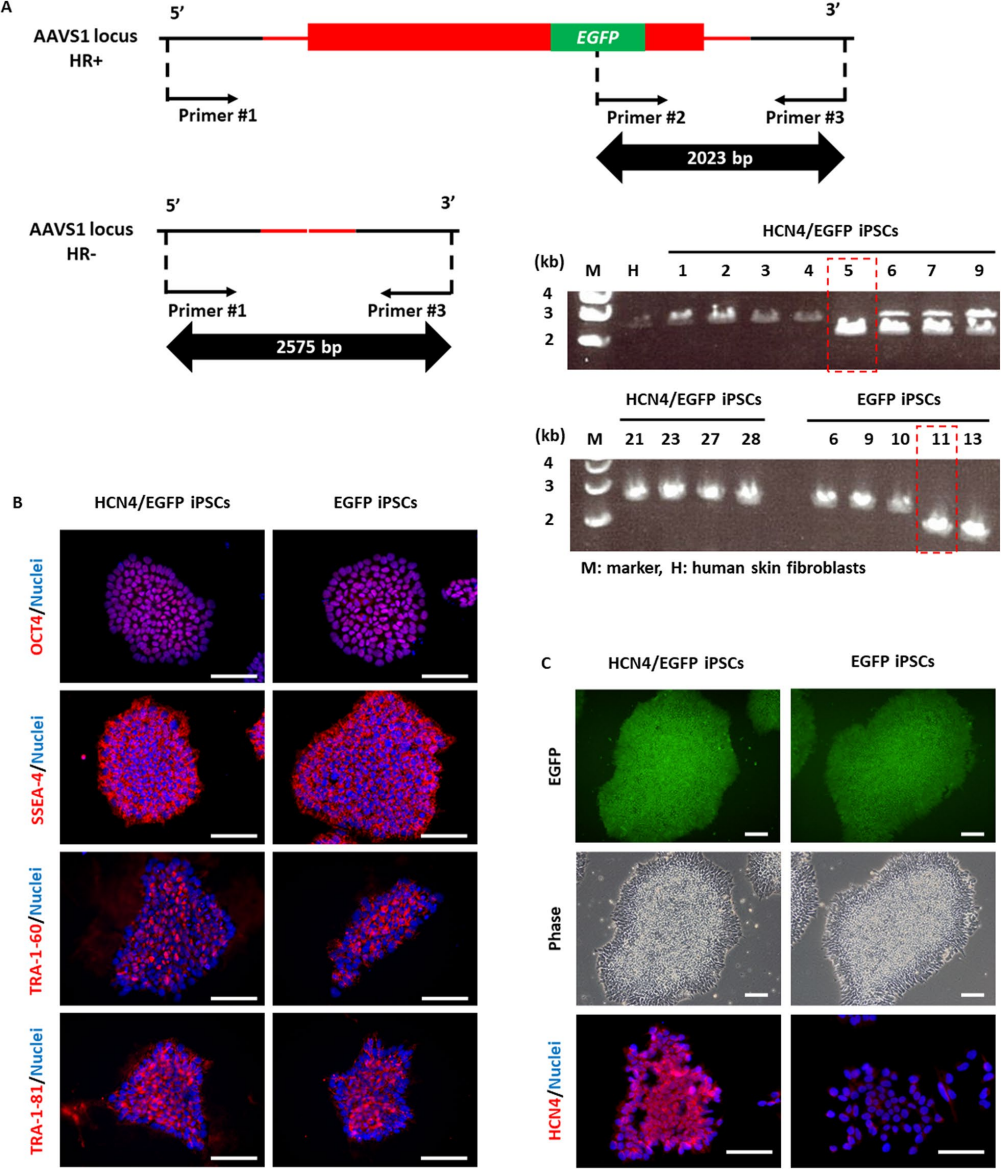
Generation of HCN4-overexpressing human induced pluripotent stem cells (iPSCs). **A** Confirmation of transgene integration in the AAVS1 locus by PCR. The 2023-bp product means homologous recombination (HR) and the 2575-bp product means random integration. Clone #5 of HCN4/EGFP iPSCs and clone #11 of EGFP iPSCs were mainly used in this study. **B** Undifferentiated markers expressed in HCN4-overexpressing and non-overexpressing iPSCs. **C** EGFP and HCN4 expression in HCN4-overexpressing and non-overexpressing iPSCs. Scale bar is 100 µm

Human *HCN4* ORF was inserted with TALEN in the AAVS1 region, where the transgene is less susceptible to silencing during differentiation [22, 23]. Homologous recombination into the AAVS1 region was confirmed by PCR (Fig. 2A). Despite overexpression of HCN4, iPS cells expressed undifferentiated markers (OCT4, SSEA-4, TRA-1-60 and TRA-1-81) (Fig. 2B) and showed a typical morphology (Fig. 2C).

### Induction of HCN4-overexpressing cardiomyocytes

**Fig. 3:**
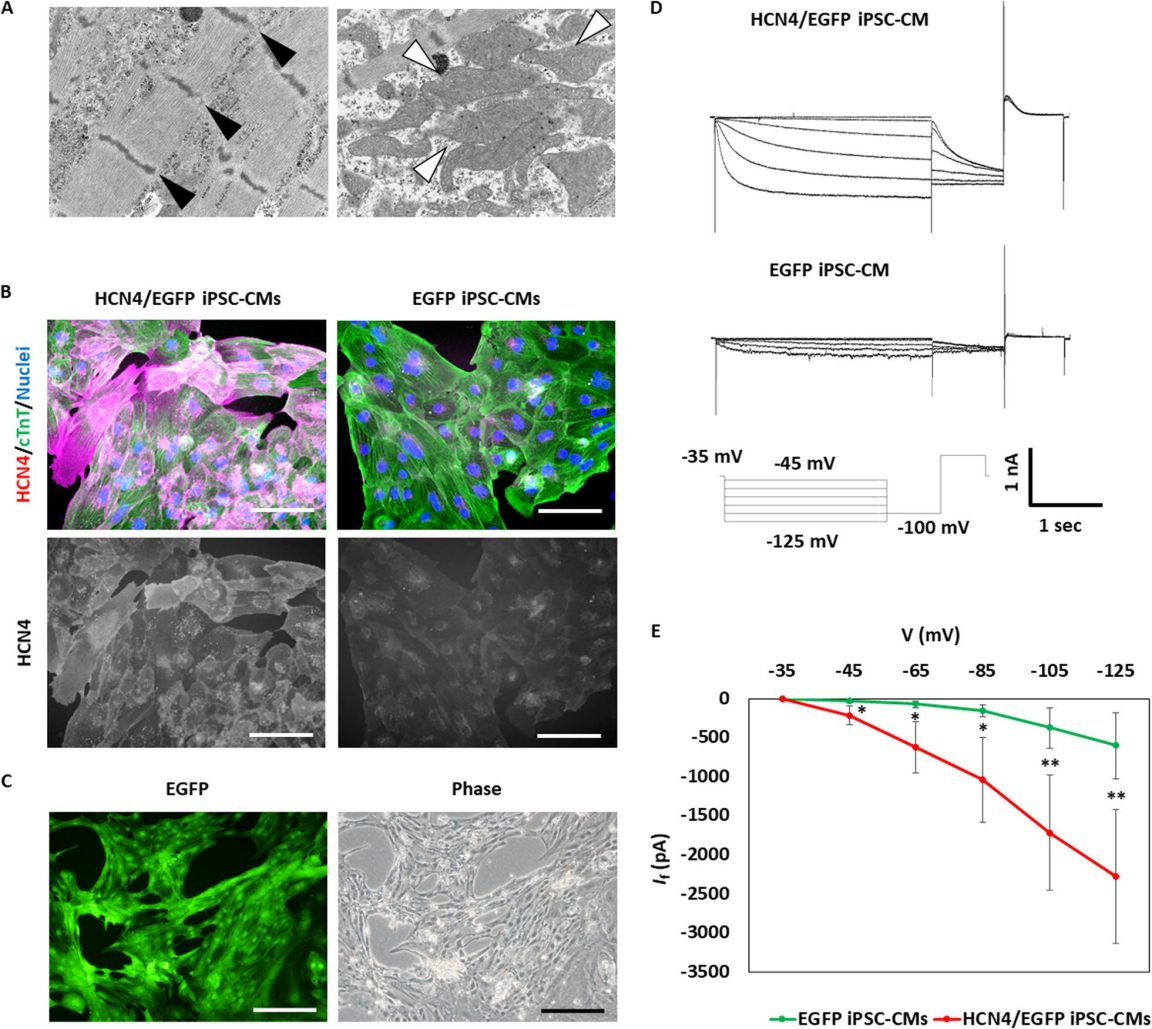
Induction of HCN4-overexpressing human induced pluripotent stem cell-derived cardiomyocytes (iPSC-CMs). **A** Transmission electron microscopy (TEM) at high magnification showed aligned Z-bands (black arrowheads) and mitochondria (white arrowheads) in HCN4-overexpressing iPSC-CMs. **B** HCN4 (magenta) and cardiac troponin T (cTnT, green) expression in iPSC-CMs on differentiation day 30. Scale bar is 100 µm. **C** EGFP expression in HCN4-overexpressing iPSC-CMs on differentiation day 103. Scale bar is 200 µm. **D** Representative *I*_f_ currents in HCN4-overexpressing (upper) or non- overexpressing (bottom) iPSC-CMs. **E** *I*_f_ − V relationship curves in HCN4-overexpressing (red line, *n* = 6) or non-overexpressing (green line, *n* = 4) iPSC-CMs (The data are shown as means ± standard deviation. *P < 0.05, **P < 0.01)

Cardiomyocytes were efficiently induced from HCN4-overexpressing iPSCs with a commonly used cardiac differentiation protocol using a GSK3 inhibitor and a Wnt inhibitor (Additional file 1). Electron micrographs showed muscle fibers and mitochondria, which were consistent with cardiomyocytes (Fig. 3A). Overexpressed HCN4 protein was confirmed by immunostaining (Fig. 3B). EGFP expression indicating transgene expression was detected in cardiomyocytes 100 days after differentiation (Fig. 3C, (Additional files 2 and 3). Enhancement of the *I*_f_ current was also confirmed by the patch clamp technique (Fig. 3D and E).

### Gene expression profile of HCN4-overexpressing cardiomyocytes

**Fig. 4:**
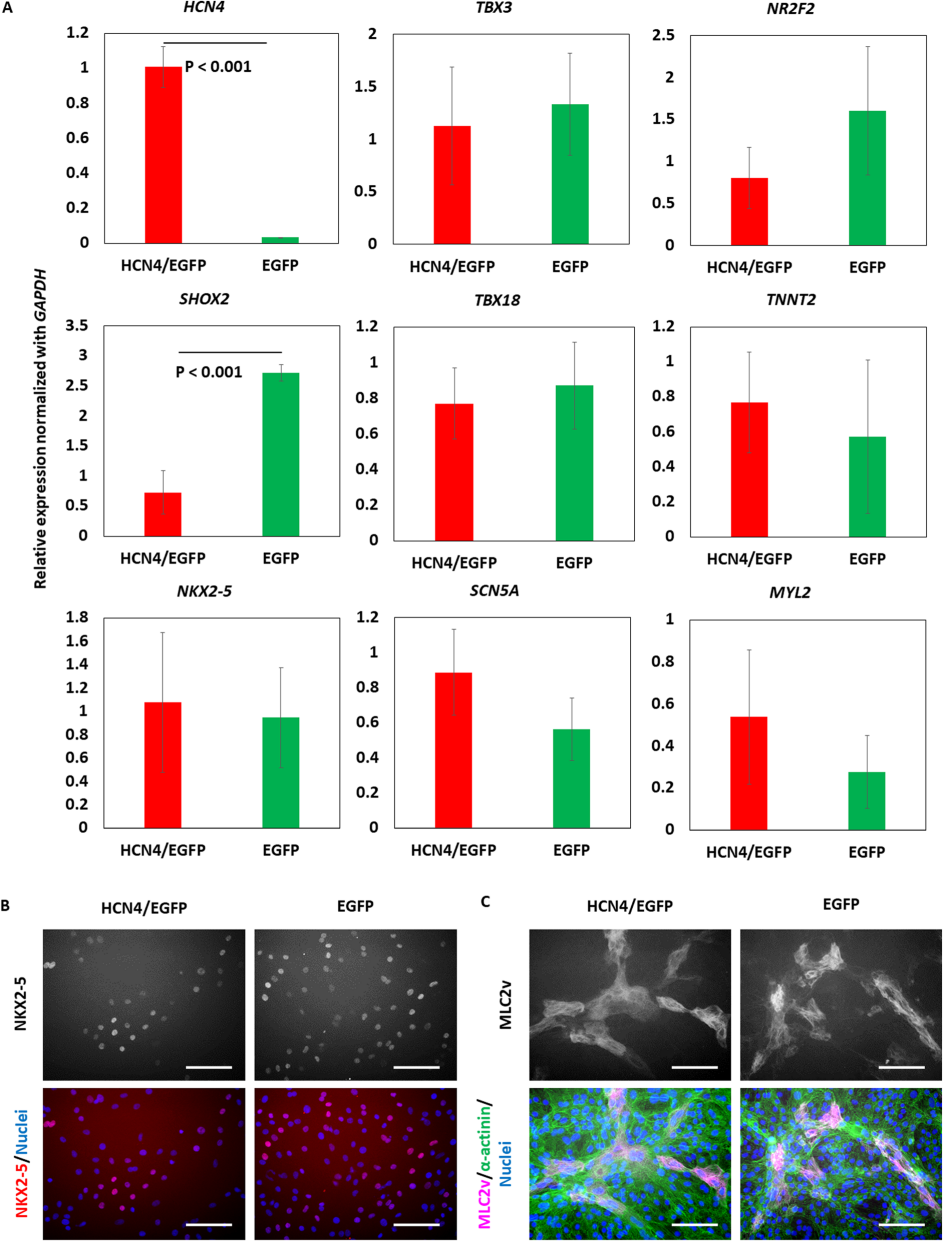
Gene expression profile of HCN4-overexpressing human induced pluripotent stem cell-derived cardiomyocytes (iPSC-CMs). **A** Messenger RNA levels evaluated by quantitative polymerase chain reaction on differentiation day 30 (*n* = 4 in each) (The data are shown as means ± standard deviation.). **B** NKX2-5 expression evaluated by immunostaining on day 35. **C** MLC2v expression evaluated by immunostaining on day 35. Scale bar is 100 µm

HCN4 mRNA level in HCN4-overexpressing iPSC-CMs was 30-times higher than that in non-overexpressing iPSC-CMs. There was no significant difference in mRNA levels of *MYL2*, a myosin isoform expressed in the ventricle, *NKX2-5*, a transcription factor expressed in the ventricle and atria, and *SCN5A*, a sodium channel expressed in the ventricle and atria. Additionally, sinoatrial node markers (*TBX3*, *TBX18*, *SHOX2*) were not upregulated in HCN4-overexpressing iPSC-CMs (Fig. 4A). Protein expression of NKX2-5 and MLC2v was also checked with immunostaining (Fig. 4B).

### Enhancement of spontaneous firing in HCN4-overexpressing cardiomyocytes

**Fig. 5:**
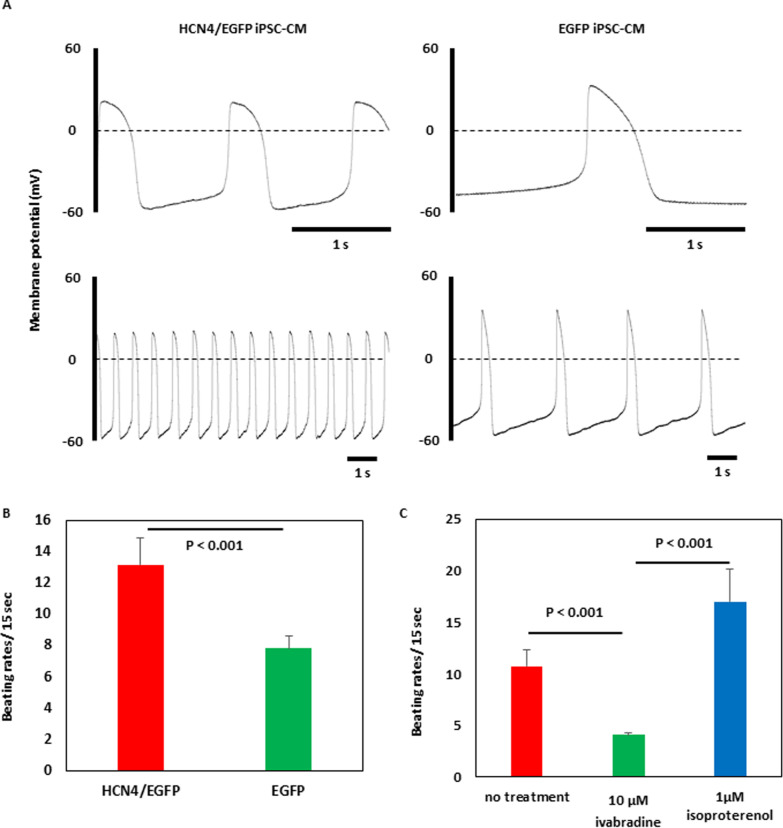
Spontaneous firing and beating rates in human induced pluripotent stem cell-derived cardiomyocytes (iPSC-CMs). **A** Action potential configurations in HCN4-overexpressing (left) and non-overexpressing (right) iPSC-CMs. **B** Comparison of spontaneous beating rates in HCN4-overexpressing and non-overexpressing iPSC-CMs (*n* = 6 in each) (The data are shown as means ± standard deviation.). **C** Responses to 10 µmol/L ivabradine and 1 µmol/L isoproterenol in HCN4-overexpressing iPSC-CMs (*n* = 8 in each) (The data are shown as means ± standard deviation)

HCN4-overexpressing cardiomyocytes showed a significantly higher rates of spontaneous firing and beating than that in non-overexpressing cardiomyocytes: 13.2 ± 1.7/15 s versus 7.8 ± 0.8/15 s (Fig. 5A and B, (Additional files 4 and 5). Additionally, the frequency of spontaneous contraction was suppressed by ivabradine and was promoted by isoproterenol as shown in Fig. 5C: control, 10.8 ± 1.3/15 s ((Additional file 6); 10 µmol/L ivabradine, 4.1 ± 0.4/15 s (Additional file 7); 1 µmol/L isoproterenol, 17.0 ± 1.8/15 s (Additional file 8).

### Enhanced pacing function by HCN4-overexpressing cardiomyocytes

**Fig. 6:**
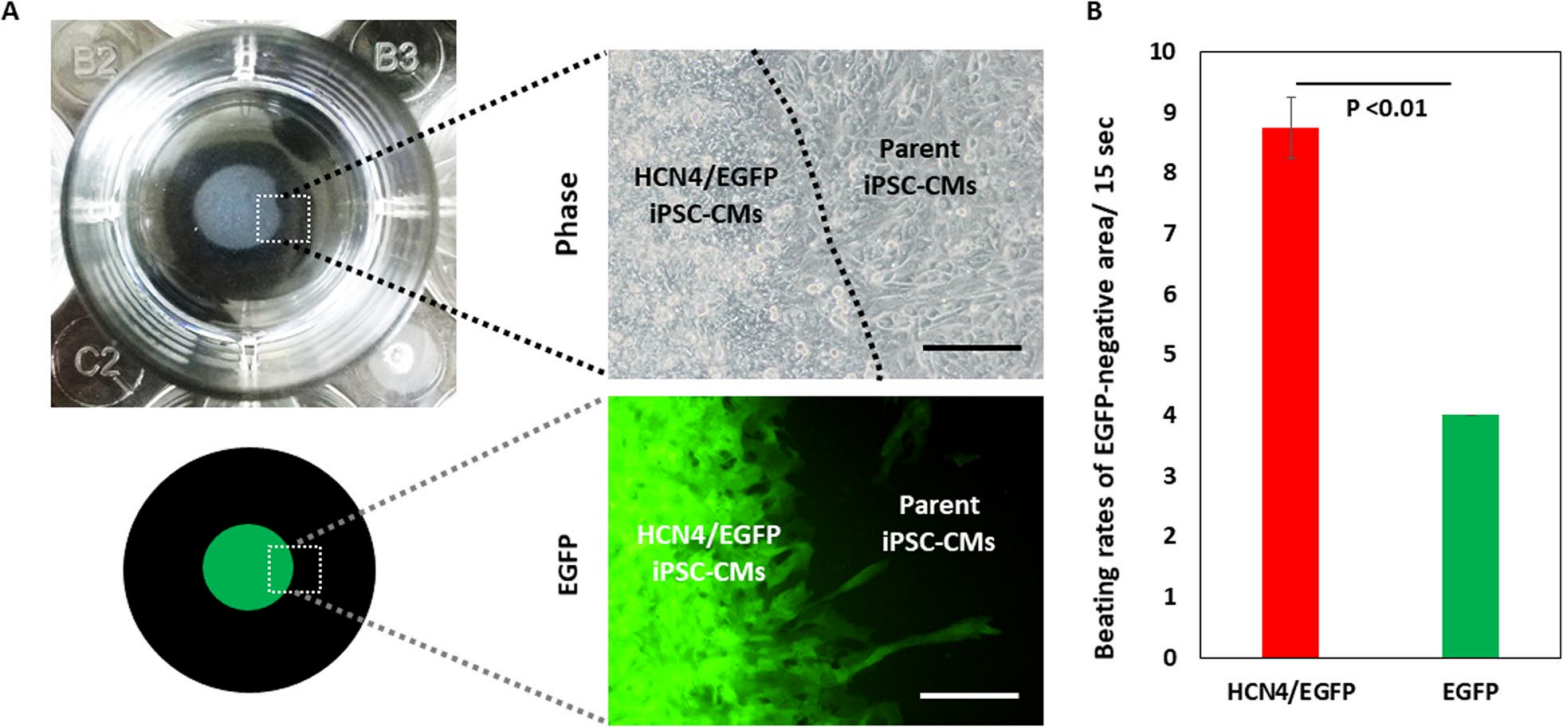
Evaluation of pacing function in vitro. **A** Macroscopic (left) and microscopic (right) images of co-culture with parent line iPSC-CMs. Scale bar is 200 µm. **B** Beating rates of EGFP-negative parent iPSC-CMs paced by EGFP-positive HCN4-overexpressing or non-overexpressing iPSC-CMs (*n* = 4 in each) (The data are shown as means ± standard deviation.)

To examine the pacing ability, HCN4-overexpressing or non-overexpressing EGFP-positive cardiomyocytes were cocultured with EGFP-negative cardiomyocytes from the parent iPSC line (Fig. 6A). EGFP-negative cardiomyocytes cocultured with HCN4-overexpressing cardiomyocytes showed a significantly higher beating frequency than that in EGFP-negative cardiomyocytes cocultured with non-overexpressing cardiomyocytes: 8.8 ± 0.5/15 s versus 4.0 ± 0.0/15 s (Fig. 6B, Additional files 9 and 10).

## Discussion

In this study, we generated HCN4-overexpressing cardiomyocytes from human iPSCs, and showed that HCN4 overexpression in human cardiomyocytes evoked a higher spontaneous firing frequency than that of control cardiomyocytes, resulting in enhanced pacing function in vitro. PSC-CMs are immature and lack *I*_k1_ current to maintain quiescent membrane potential and have a relatively high maximal diastolic potential (− 60 mV), which may not be sufficient to activate HCN channels [24]. However, a significant increase in *I*_f_ was observed in HCN4-overexpressing cardiomyocytes at − 45 and − 65 mV as shown in Fig. 3E. In addition, the spontaneous contraction frequency was suppressed by the HCN channel inhibitor ivabradine (Fig. 5C), suggesting that the enhancement of diastolic depolarization by increased *I*_f_ current caused by HCN4 overexpression led to an enhanced pacing effect of iPSC-CMs. So far, many groups, including us, have reported an increase in the frequency of sponteneous firing and the addition of a pacing function by transduction of HCN genes in mouse ESC-CMs, mesenchymal stem cells or the left ventricle [11, 25–32]. Results in this study are consistent with the findings of previous studies.

As previously reported [23, 33], it was possible to confirm long-term expression not only in iPSCs but also in iPSC-CMs by transduction of *HCN4* into the AAVS1 safe harbor locus. We have also experienced loss of EGFP expression during differentiation in cardiomyocytes and non-cardiomyocytes derived from EGFP-positive iPSC lines without homologous recombination of the transgene (data was not shown).

HCN4-overexpressing cardiomyocytes were induced very efficiently since the commonly used efficient cardiac differentiation protocol using a GSK3 inhibitor and a Wnt inhibitor and the cardiomyocyte purification method can be applied [20, 21, 34]. Therefore, our strategy for generating cardiomyocytes with enhanced pacing function is simple and feasible compared to previously reported methods for generating sinoatrial node-like cells from human PSCs using complicated combinations of growth factors and compounds [4–8].

In the embryonic heart, HCN4 expression is first seen in the cardiac crescent, the source of the left ventricle and atria, and as it develops, it becomes localized to the conduction system, including the sinoatrial node [35, 36]. In our study, there was no significant upregulation of sinoatrial node marker genes or working myocyte marker genes in HCN4-overexpressing iPSC-CMs as far as we examined with qPCR. This suggests that constitutive HCN4 overexpression did not change the cardiomyocyte subtype into sinoatrial node-like cells. The proportions of ventricular, atrial and nodal type in human PSC-CMs vary depending on the differentiation protocols and cell lines [20, 37, 38]. That is, the baseline cardiomyocyte phenotype might affect the spontaneous firing frequency and pacing function also when HCN4 is overexpressed. For better integration between transplanted cells and the recipient myocardium, HCN4-overexpressing ventricular myocytes might be useful for transplantation into the ventricle, while HCN4-overexpressing atrial myocytes might be better for transplantation into the atria.

### Study limitations

Only in vitro studies have been conducted. It has not been investigated how much pacing can be done in vivo. Since the connexin expression patterns are different between the atrium and ventricles or between healthy and diseased hearts [39], the pacing ability in vivo might be affected by various conditions.

The firing frequency probably depends on the expression level of transduced HCN4. Although EGFP expression was detected for more than 3 months in vitro in this study, the maintenance of transgene expression over a longer period should be checked, especially in vivo.

In addition, the possibility of tachycardia is a concern because some groups have reported that transplantation of a large number of pluripotent stem cell-derived cardiomyocytes transplanted into an infarcted heart resulted in ventricular tachyarrhythmias [40–42]. However, it might be possible to control the pacing rate with drugs because spontaneous firing responded to an HCN channel inhibitor, ivabradine, in this study. Actually, Nakamura et al. also showed an anti-arrhythmic effect of ivabradine on iPSC-CM engraftment arrhythmias [43].

Cardiac Troponin I promoter-driven human HCN4 transgenic mice showed similar cardiac morphology at birth but cardiac dilatation after birth due to dysregulation of calcium homeostasis and increased myocardial apoptosis [44]. Therefore, it is necessary to investigate the effect of long-term HCN4 overexpression on transplanted cells in vivo in the future.

Small animals such as mice and rats have a faster heart rate than humans (> 350 bpm). Even if atrioventricular block is performed to create a bradycardia model, its heart rate is more than 120 bpm (Saito et al. Int Heart J. 2018), making it difficult to evaluate the pacing function of human cells. Therefore, in vivo studies need to use large animals such as monkeys or pigs that have been treated with immunosuppressive drugs. In addition, tumorigenic potential should be assessed.

## Conclusions

We generated HCN4-overexpressing human iPSC-CMs showing enhancement of *I*_f_ current and pacing ability. Application of these cells with added pacing function to a biological pacemaker is expected in the future.

## Supplementary Information

**Additional file 1**: Differentiation of day 8 cardiomyocytes derived from HCN4-overexpressing human induced pluripotent stem cells.

**Additional file 2**: HCN4-overexpressing cardiomyocytes on differentiation day 103 (GFP).

**Additional file 3**: HCN4-overexpressing cardiomyocytes on differentiation day 103 (phase contrast).

**Additional file 4**: HCN4-overexpressing cardiomyocytes on day 27

**Additional file 5**: Non-overexpressing cardiomyocytes on day 27.

**Additional file 6:** Drug response of HCN4-overexpressing cardiomyocytes (no treatment).

**Additional file 7**: Drug response of HCN4-overexpressing cardiomyocytes (10 µmol/L ivabradine).

**Additional file 8**: Drug response of HCN4-overexpressing cardiomyocytes (1 µmol/L isoproterenol).

**Additional file 9**: Parent cardiomyocytes paced by HCN4-overexpressing cardiomyocytes. The left half is HCN4-overexpressing cardiomyocytes and the right half is parental line-derived cardiomyocytes.

**Additional file 10**: Parent cardiomyocytes paced by non-overexpressing cardiomyocytes. The left half is non-overexpressing cardiomyocytes and the right half is parental line-derived cardiomyocytes.

## Data Availability

The datasets used and analyzed during the current study are available from the corresponding author on reasonable request.

## Abbreviations

CAR: Chimeric antigen receptor
cTnT: Cardiac Troponin T
ESCs: Embryonic stem cells
ESC-CMs: Embryonic stem cell-derived cardiomyocytes
HCN channel: Hyperpolarization-activated cyclic nucleotide-gated potassium channel
iPSCs: Induced pluripotent stem cells
iPSC-CMs: Induced pluripotent stem cell-derived cardiomyocytes
PCR: Polymerase chain reaction
qPCR: Quantitative polymerase chain reaction
PSCs: Pluripotent stem cells
PSC-CMs: Pluripotent stem cell-derived cardiomyocytes
TEM: Transmission electron microscopy
